# Trends in Diversity and Representativeness of Health Care Workers in the United States, 2000 to 2019

**DOI:** 10.1001/jamanetworkopen.2021.17086

**Published:** 2021-07-15

**Authors:** Dan P. Ly, Anupam B. Jena

**Affiliations:** 1 Division of General Internal Medicine and Health Services Research, David Geffen School of Medicine at the University of California, Los Angeles; 2 VA Greater Los Angeles Healthcare System, Los Angeles, California; 3 Department of Health Care Policy, Harvard Medical School, Boston, Massachusetts; 4 Massachusetts General Hospital, Boston; 5 National Bureau of Economic Research, Cambridge, Massachusetts

## Abstract

This cross-sectional study examines national trends in representativeness in select health care occupations by race/ethnicity and sex.

## Introduction

Despite evidence that greater diversity among health care workers may allow them to better care for patients with diverse cultural, social, economic, and clinical needs,^1,2,3^ data are limited on trends in diversity and representativeness of US health care workers by occupation. Using US Census data from 2000 to 2019, we examined national trends in representativeness in select health care occupations by race/ethnicity and sex.

## Methods

In this cross-sectional study, we used 2 data sources: (1) the Decennial Census long form, a nationally representative, US Census–administered survey, from 2000, and (2) the American Community Survey, a nationally representative Census-administered survey, from 2001 to 2019. Response rates for both exceeded 90%.^4,5^ This study followed the Strengthening the Reporting of Observational Studies in Epidemiology (STROBE) reporting guideline. The University of California Los Angeles institutional review board determined that the study was not human subjects research and therefore exempt from review and the requirement for informed consent.

Occupation, race, (White, Black, American Indian or Alaska Native, Chinese, Japanese, other Asian or Pacific Islander, other race, 2 major races, or ≥3 major races) and ethnicity (not Hispanic, Mexican, Puerto Rican, Cuban, and other Hispanic origin) were self-reported. Participants who selected Chinese, Japanese, or other Asian or Pacific Islander were included into the category Asian. Participants who selected Mexican, Puerto Rican, Cuban, and other were included in the Hispanic category. Participants who selected other race, 2 major races, or 3 or more major races were excluded, leaving 4 groups: non-Hispanic White, non-Hispanic Black, non-Hispanic Asian, and Hispanic. We estimated the composition of select health care occupations (Table 1) by race/ethnicity and sex in 2000 to 2004 and 2015 to 2019. For each occupation, we calculated the change in percentage in each race/ethnicity and sex subgroup between these 2 periods using linear regression. In addition, in each occupation, we assessed the population representativeness of each race/ethnicity and sex subgroup by calculating the ratio of the percentage of a specific subgroup in an occupation (eg, Black male physicians) to the percentage of the US population in that subgroup (eg, Black men).^6^ We used Census-provided sampling weights to make nationally representative estimates. *P* values were from 2-sided tests, and results were deemed statistically significant at *P* < .05. Analyses were conducted in Stata version 16.1 (StataCorp).

**Table 1.** Trends in Racial and Ethnic Diversity of Select Health Care Occupations, 2000 to 2019^a^

Subgroup	Health care workers, % (95% CI)		Difference (95% CI), percentage points
	2000-2004	2015-2019	
**Physicians and surgeons**			
White men	54.1 (53.3 to 54.9)	43.8 (43.3 to 44.3)	−10.3 (−11.2 to −9.3)
White women	18.6 (18.0 to 19.2)	21.6 (21.2 to 22.0)	3.0 (2.3 to 3.7)
Black men	2.6 (2.3 to 2.9)	2.5 (2.3 to 2.7)	−0.1 (−0.5 to 0.2)
Black women	2.0 (1.8 to 2.3)	2.6 (2.4 to 2.8)	0.6 (0.2 to 0.9)
Hispanic men	3.7 (3.3 to 4.0)	3.9 (3.7 to 4.1)	0.2 (−0.2 to 0.6)
Hispanic women	1.6 (1.4 to 1.8)	2.5 (2.3 to 2.7)	0.9 (0.6 to 1.2)
Asian men	10.3 (9.8 to 10.8)	11.8 (11.5 to 12.1)	1.5 (0.9 to 2.1)
Asian women	5.6 (5.3 to 6.0)	8.9 (8.6 to 9.2)	3.2 (2.7 to 3.7)
**Pharmacists**			
White men	43.6 (42.3 to 45.0)	31.5 (30.7 to 32.3)	−12.2 (−13.7 to −10.6)
White women	34.5 (33.3 to 35.8)	36.6 (35.7 to 37.4)	2.0 (0.5 to 3.5)
Black men	2.4 (1.8 to 3.1)	2.3 (2.0 to 2.7)	−0.06 (−0.8 to 0.6)
Black women	2.8 (2.2 to 3.4)	4.0 (3.6 to 4.5)	1.3 (0.6 to 2.0)
Hispanic men	1.9 (1.5 to 2.5)	1.6 (1.4 to 1.9)	−0.3 (−0.9 to 0.2)
Hispanic women	1.7 (1.4 to 2.2)	2.6 (2.3 to 2.9)	0.9 (0.3 to 1.4)
Asian men	4.8 (4.2 to 5.5)	7.4 (6.9 to 7.9)	2.5 (1.7 to 3.4)
Asian women	7.0 (6.3 to 7.9)	11.8 (11.2 to 12.5)	4.8 (3.8 to 5.8)
**Therapists**			
White men	16.9 (16.2 to 17.6)	14.3 (13.9 to 14.7)	−2.6 (−3.4 to −1.8)
White women	66.5 (65.6 to 67.4)	61.9 (61.4 to 62.5)	−4.6 (−5.6 to −3.5)
Black men	1.9 (1.6 to 2.3)	1.8 (1.6 to 1.9)	−0.2 (−0.6 to 0.2)
Black women	4.5 (4.1 to 5.0)	5.3 (5.0 to 5.6)	0.8 (0.3 to 1.3)
Hispanic men	1.1 (0.9 to 1.3)	1.9 (1.8 to 2.1)	0.9 (0.6 to 1.1)
Hispanic women	3.2 (2.8 to 3.6)	5.7 (5.4 to 6.0)	2.5 (2.0 to 3.0)
Asian men	1.7 (1.4 to 2.0)	2.4 (2.2 to 2.6)	0.7 (0.4 to 1.0)
Asian women	3.0 (2.6 to 3.3)	4.6 (4.3 to 4.8)	1.6 (1.2 to 2.0)
**Nurses**			
White men	5.8 (5.6 to 6.0)	6.7 (6.5 to 6.8)	0.9 (0.7 to 1.1)
White women	73.8 (73.4 to 74.3)	64.6 (64.3 to 64.9)	−9.2 (−9.7 to −8.7)
Black men	0.6 (0.6 to 0.7)	1.2 (1.2 to 1.3)	0.6 (0.5 to 0.7)
Black women	8.1 (7.8 to 8.4)	9.6 (9.4 to 9.8)	1.5 (1.2 to 1.8)
Hispanic men	0.5 (0.4 to 0.6)	1.1 (1.0 to 1.2)	0.6 (0.5 to 0.7)
Hispanic women	3.1 (3.0 to 3.3)	5.8 (5.6 to 5.9)	2.6 (2.4 to 2.8)
Asian men	0.7 (0.6 to 0.8)	1.5 (1.4 to 1.6)	0.8 (0.7 to 0.9)
Asian women	5.9 (5.7 to 6.1)	7.3 (7.2 to 7.5)	1.4 (1.2 to 1.7)
**Dentists**			
White men	69.8 (68.2 to 71.4)	54.2 (53.0 to 55.4)	−15.6 (−17.6 to −13.5)
White women	11.6 (10.6 to 12.7)	17.7 (16.8 to 18.6)	6.1 (4.7 to 7.5)
Black men	2.1 (1.6 to 2.7)	1.5 (1.3 to 1.9)	−0.5 (−1.1 to 0.1)
Black women	1.0 (0.7 to 1.4)	1.7 (1.4 to 2.2)	0.7 (0.2 to 1.3)
Hispanic men	2.5 (2.0 to 3.1)	3.3 (2.9 to 3.8)	0.8 (0.07 to 1.6)
Hispanic women	1.6 (1.2 to 2.2)	2.7 (2.3 to 3.1)	1.0 (0.4 to 1.7)
Asian men	6.2 (5.4 to 7.1)	8.9 (8.2 to 9.7)	2.7 (1.6 to 3.9)
Asian women	3.9 (3.3 to 4.7)	7.9 (7.3 to 8.6)	4.0 (3.0 to 5.0)
**Health care aide**			
White men	5.9 (5.7 to 6.2)	5.1 (4.9 to 5.2)	−0.8 (−1.1 to −0.6)
White women	45.4 (44.9 to 46.0)	37.9 (37.5 to 38.2)	−7.6 (−8.2 to −7.0)
Black men	3.4 (3.2 to 3.7)	3.7 (3.5 to 3.9)	0.2 (−0.03 to 0.5)
Black women	27.8 (27.3 to 28.3)	29.1 (28.8 to 29.5)	1.3 (0.7 to 2.0)
Hispanic men	1.3 (1.2 to 1.4)	1.9 (1.8 to 2.0)	0.6 (0.4 to 0.7)
Hispanic women	10.0 (9.7 to 10.4)	13.9 (13.6 to 14.2)	3.9 (3.4 to 4.3)
Asian men	0.7 (0.7 to 0.8)	1.2 (1.1 to 1.2)	0.4 (0.3 to 0.5)
Asian women	2.7 (2.5 to 2.9)	4.2 (4.0 to 4.3)	1.5 (1.2 to 1.7)

^a^ Authors’ calculation using the Decennial Census in 2000 and the American Community Survey data from 2001 to 2019. Results were weighted using Census-provided sampling weights to represent the US population. Occupation and race were self-reported. Nurses include those identifying themselves as registered nurses and do not include nurse practitioners. Pharmacists and dentists include those identifying themselves as such. Therapists include occupational therapists, physical therapists, respiratory therapists, and speech language pathologists. Health care aides include those identifying themselves as nursing, psychiatric, and home health aides. Individuals identifying as other race/ethnicity were not included.

## Results

 Our study included 1 648 924 individuals (1 303 496 [79.1%] women; 345 428 [20.9%] men) across 6 health care occupations (Table 1). The percentage of White men in relatively more remunerative occupations (ie, physicians and surgeons, pharmacists, and dentists) decreased between 2000 to 2004 and 2015 to 2019. For example, the percentage of physicians and surgeons who were White men decreased from 54.1% (95% CI, 53.3% to 54.9%) to 43.8% (95% CI, 43.3% to 44.3%) (difference, −10.3 [95% CI, −11.2 to −9.3] percentage points), while the percentage of dentists who were White men decreased 15.6 (95% CI, 13.5 to 17.6) percentage points. Little to no change was observed in the percentage of men from underrepresented minorities in these occupations. For example, no change was observed in the percentage of physicians and surgeons who were Hispanic men (difference, 0.2 [95% CI, −0.2 to 0.6] percentage points) or in the percentage of pharmacists who were Black men (difference, −0.06 [95% CI, −0.8 to 0.6] percentage points). Statistically significant increases were observed in the percentage of both Black and Hispanic women in these 3 occupations. For example, the percentage of pharmacists who were Black women increased 1.3 (95% CI, 0.6 to 2.0) percentage points, and the percentage of dentists who were Hispanic women increased 1.0 (95% CI, 0.4 to 1.7) percentage points. Increases for White and Asian women in these occupations were larger than for Black and Hispanic women.

Significant increases were observed among most minority race/ethnicity and sex subgroups in less remunerative occupations (ie, nurses, therapists, and health care aides). In an analysis of population representativeness in 2015 to 2019, Black and Hispanic men and women remained considerably underrepresented relative to the US population in more remunerative occupations (Table 2).

**Table 2.** Representativeness of Select Health Care Occupations in 2015-2019 Period^a^

Subgroup	Health care workers, % (95% CI)	Total population, % (95% CI)	Ratio
**Physicians and surgeons**			
White men	43.8 (43.3-44.3)	29.9 (29.9-30.0)	1.46
White women	21.6 (21.2-22.0)	30.8 (30.7-30.8)	0.70
Black men	2.5 (2.3-2.7)	5.9 (5.9-5.9)	0.42
Black women	2.6 (2.4-2.8)	6.5 (6.4-6.5)	0.40
Hispanic men	3.9 (3.7-4.1)	9.1 (9.1-9.1)	0.43
Hispanic women	2.5 (2.3-2.7)	8.9 (8.9-9.0)	0.28
Asian men	11.8 (11.5-12.1)	2.7 (2.6-2.7)	4.44
Asian women	8.9 (8.6-9.2)	3.0 (2.9-3.0)	3.00
**Pharmacists**			
White men	31.5 (30.7-32.3)	29.9 (29.9-30.0)	1.05
White women	36.6 (35.7-37.4)	30.8 (30.7-30.8)	1.19
Black men	2.3 (2.0-2.7)	5.9 (5.9-5.9)	0.39
Black women	4.0 (3.6-4.5)	6.5 (6.4-6.5)	0.62
Hispanic men	1.6 (1.4-1.9)	9.1 (9.1-9.1)	0.18
Hispanic women	2.6 (2.3-2.9)	8.9 (8.9-9.0)	0.29
Asian men	7.4 (6.9-7.9)	2.7 (2.6-2.7)	2.77
Asian women	11.8 (11.2-12.5)	3.0 (2.9-3.0)	4.01
**Therapists**			
White men	14.3 (13.9-14.7)	29.9 (29.9-30.0)	0.48
White women	61.9 (61.4-62.5)	30.8 (30.7-30.8)	2.01
Black men	1.8 (1.6-1.9)	5.9 (5.9-5.9)	0.30
Black women	5.3 (5.0-5.6)	6.5 (6.4-6.5)	0.82
Hispanic men	1.9 (1.8-2.1)	9.1 (9.1-9.1)	0.21
Hispanic women	5.7 (5.4-6.0)	8.9 (8.9-9.0)	0.64
Asian men	2.4 (2.2-2.6)	2.7 (2.6-2.7)	0.91
Asian women	4.6 (4.3-4.8)	3.0 (2.9-3.0)	1.55
**Nurses**			
White men	6.7 (6.5-6.8)	29.9 (29.9-30.0)	0.22
White women	64.6 (64.3-64.9)	30.8 (30.7-30.8)	2.10
Black men	1.2 (1.2-1.3)	5.9 (5.9-5.9)	0.21
Black women	9.6 (9.4-9.8)	6.5 (6.4-6.5)	1.48
Hispanic men	1.1 (1.0-1.2)	9.1 (9.1-9.1)	0.12
Hispanic women	5.8 (5.6-5.9)	8.9 (8.9-9.0)	0.64
Asian men	1.5 (1.4-1.6)	2.7 (2.6-2.7)	0.56
Asian women	7.3 (7.2-7.5)	3.0 (2.9-3.0)	2.48
**Dentists**			
White men	54.2 (53.0-55.4)	29.9 (29.9-30.0)	1.81
White women	17.7 (16.8-18.6)	30.8 (30.7-30.8)	0.58
Black men	1.5 (1.3-1.9)	5.9 (5.9-5.9)	0.26
Black women	1.7 (1.4-2.2)	6.5 (6.4-6.5)	0.27
Hispanic men	3.3 (2.9-3.8)	9.1 (9.1-9.1)	0.36
Hispanic women	2.7 (2.3-3.1)	8.9 (8.9-9.0)	0.30
Asian men	8.9 (8.2-9.7)	2.7 (2.6-2.7)	3.36
Asian women	7.9 (7.3-8.6)	3.0 (2.9-3.0)	2.68
**Health care aide**			
White men	5.1 (4.9-5.2)	29.9 (29.9-30.0)	0.17
White women	37.9 (37.5-38.2)	30.8 (30.7-30.8)	1.23
Black men	3.7 (3.5-3.9)	5.9 (5.9-5.9)	0.62
Black women	29.1 (28.8-29.5)	6.5 (6.4-6.5)	4.52
Hispanic men	1.9 (1.8-2.0)	9.1 (9.1-9.1)	0.21
Hispanic women	13.9 (13.6-14.2)	8.9 (8.9-9.0)	1.56
Asian men	1.2 (1.1-1.2)	2.7 (2.6-2.7)	0.43
Asian women	4.2 (4.0-4.3)	3.0 (2.9-3.0)	1.41

^a^ Authors’ calculation using the American Community Survey data from 2015 to 2019. Ratio is the ratio of percentage in occupation to percentage in U.S. population in 2015 to 2019. Results were weighted using Census-provided sampling weights to represent the US population. Occupation and race were self-reported. Nurses include those identifying themselves as registered nurses and do not include nurse practitioners. Pharmacists and dentists include those identifying themselves as such. Therapists include occupational therapists, physical therapists, respiratory therapists, and speech language pathologists. Health care aides include those identifying themselves as nursing, psychiatric, and home health aides. Individuals identifying as other race/ethnicity were not included.

## Discussion

In a nationally representative sample, little to no increase in Black or Hispanic men was observed among physicians and surgeons, pharmacists, and dentists between 2000 and 2019. While there were increases in Black and Hispanic women in these occupations, increases among White and Asian women were larger. Altogether, increases in representation of these 4 subgroups of women were accompanied by large declines in the proportion of White men. Other health care occupations, such as nurses, therapists, and health care aides, had increases across most minority subgroups during the examined period. These results quantify the current representativeness of the US health care workforce and changes in its composition during the past 2 decades. Study limitations include the use of self-reported survey data.
